# Effects of increased levels of atmospheric CO_2_ and high temperatures on rice growth and quality

**DOI:** 10.1371/journal.pone.0187724

**Published:** 2017-11-16

**Authors:** Shuo Liu, Muhammad Ahmed Waqas, Song-he Wang, Xiang-yang Xiong, Yun-fan Wan

**Affiliations:** 1 Key Laboratory of Agricultural Environment, Institute of Environment and Sustainable Development in Agriculture, Chinese Academy of Agricultural Sciences, Beijing, China; 2 Department of Agriculture, Yangtze University, Jingzhou, Hubei Province, China; International Rice Research Institute, PHILIPPINES

## Abstract

The increased atmospheric temperatures resulting from the increased concentration of atmospheric carbon dioxide (CO_2_) have had a profound influence on global rice production. China serves as an important area for producing and consuming rice. Therefore, exploring the effects of the simultaneously rising levels of atmospheric CO_2_ and temperatures on rice growth and quality in the future is very important. The present study was designed to measure the most important aspects of variation for rice-related physiological, ecological and quality indices in different growing periods under a simultaneous increase of CO_2_ and temperature, through simulation experiments in climate-controlled growth chambers, with southern rice as the study object. The results indicated that the ecological indices, rice phenology, and leaf area would decrease under a simultaneous increase of CO_2_ and temperature. For the physiological indices, Malondialdehyde (MDA) levels increased significantly in the seedling period. However, it showed the trend of increase and subsequent decrease in the heading and filling periods. In addition, the decomposition of soluble protein (SP) and soluble sugar (SS) accelerated in filling period. The rice quality index of the Head Rice Rate showed the decreasing trend and subsequent increase, but the Chalky Rice Rate and Protein Content indices gradually decreased while the Gel Consistency gradually increased.

## Introduction

By the end of the 21^st^ century, the atmospheric greenhouse gas CO_2_ concentration (CO_2_) is predicted to increase from the current 370 μmol·mol^−1^ to 540–970 μmol·mol^−1^, and reach 550 μmol·mol^−1^ by 2050 and 750 μmol·mol^−1^ by 2100; meanwhile, global surface temperatures will increase from approximately 2°C and 4°C compared to 1990, respectively [1]. The level of CO_2_ in the atmosphere will continue to increase so that climate warming will become the main feature of future climate change. The interaction between CO_2_ and temperature will certainly exert a profound influence on the earth’s environment and global agricultural production [2–5].

China, the largest rice producing and consuming country in the world, grows rice in areas covering 27% of all its grain crop area [6, 7]. The rice belt in Yangtze River Valley serves as the largest rice belt in China. In this area, the most important rice growing stage is mostly the high temperature period in July–August, when the temperature in this period remains above 35°C. As the global surface temperatures rise, the risk and frequency of high temperature events during this stage will be higher, which would seriously threaten food security in China in the future and necessitate relevant research.

Currently, research studies on the effects of higher CO_2_ levels and temperatures on rice have mainly focused on the effects of single factors on rice production [8–9]. Some tests have used open top climate chambers and infrared heating for free-air temperature controlled enhancement in large plots to study the effects of a simultaneous increase in atmospheric CO_2_ and temperature on rice [10–11]. However, the warming measures in those studies were implemented in divided periods of daytime or nighttime and limited by environment, operational costs and other conditions, with a relatively small test gradient. With the increasing seriousness of global warming, a multi-gradient all-day simulation test of increased atmospheric CO_2_ levels and high temperature is urgently needed for the southern rice growing region of China.

Compared to open top climate chambers and infrared heating for temperature free-air controlled enhancement, a manual climatic chamber that has a better ability to control environmental variables and has better operation procedures, is more appropriate to conduct a multi-gradient and multi-factor simulation test. The present study involved the measurement of variation in important rice physiological, ecological, and rice production quality indices in different growing periods under a simultaneous increase in CO_2_ concentrations and temperatures, using a simulation experiment and climatic chambers, with the rice varieties named Liangyou 287, which are temperature sensitive and are the prevailing hybrid varieties planted in the study area. The goal was to provide basic data and scientific reference information in support of the future scientific study, improved production measures, and policy formulation relevant to Chinese rice production in response to climate change.

## Materials and methods

### Overview of the study area

The study area, located in the Jianghan Plain, forms part of the main southern rice production area of China. Located at the Jingzhou Agricultural Experiment Station, Hubei Province (30°21′01″N, 112°09′09″E), the area has a subtropical monsoon climate, with an annual average temperature of 16.2°C, annual sunshine duration about 2000 h, total annual solar radiation about 460–480 KJ × cm^−2^, and a frost-free period of about 240–260 days. The area experiences 230–240 days above 10°C, has an active accumulated temperature 5100–5300°C, and yellow-brown paddy soil.

### Study methods

#### Experimental treatments

Combined with the results of research related to long-time-scale increases in atmospheric CO_2_ levels and high temperature in the Intergovernmental Panel on Climate Change, the present study employed a simulation test with three treatments: control (CK), moderate (M) and intensive (H) levels of both CO_2_ and temperature. Therefore, the temperature of the CK treatment was set to be the historic temperature in the 2013 rice growing season in the study area (24–29°C), and the CO_2_ in the manual climate-control chamber was the local average CO_2_ concentration (400 μmol·mol^−1^). For the M treatment temperate increased by 2°C compared with CK, and CO_2_ in the chamber was set to 550 μmol·mol^−1^. For the H treatment temperature increased by 4°C compared with CK, and CO_2_ in the chamber was set to 650 μmol·mol^−1^.

#### Selection of simulation parameters

In order to simulate the climatic conditions of the local region, the meteorological variables of the study area were analyzed as follows: (1) the standard deviation of the average value of meteorological variables was calculated for many years in the growing season of the study area, and the range of deviation was determined for each meteorological variable; (2) the meteorological variables recorded in a certain growing season were compared and selected if they the range of standard deviation determined by (1); that is, the meteorological variables thus selected for a partial growing season/year were selected as the simulation operation values.

Based on the representative time verification method described above, the changes in mean temperature, relative humidity and total sunshine duration in the rice growing season from April to September were analyzed by comparing the daily meteorological data of Jingzhou Meteorological Station in 2002–2011 and 2013 with the simulation data. Finally, the 2013 meteorological variables in the study area were found to adequately represent local conditions; therefore, the average temperature, relative humidity, and total sunshine duration of the growing season of control treatment (CK) were based on the meteorological data of the study area in 2013. That is, the meteorological variables of April 2013 were used as the CK treatment baseline for the cultivation environment of the rice seedling stage.

#### Calibration and input of simulation parameters

Before the start of the test, a 2 m wide × 4 m long × 2 m tall manually controlled temperature climatic chamber (Thermoline Scientific, Smithfield, NWS, Australia), an EE08 humidity sensor (E&E Electronic, Eigerwitzdorf, Austria) and a CMP222 CO_2_ probe (Vaisala, Helsinki, Finland) were calibrated and stability testing was conducted by a nationally accredited testing organization to ensure the reliability of the simulation and related environmental controls.

Climatic control was based on weather data for daily temperature, relative humidity, and sunshine duration recorded at 2:00, 8:00, 14:00 and 20:00 by the Jingzhou Meteorological Station in 2013. Based on this data and using the daily variation at 2 h intervals in the rice sowing stage, we controlled the conditions in each climatic chamber so as to achieve a daily gradual change in climatic conditions designed to simulate natural conditions. At the same time, the environment parameters in the chamber were automatically recorded every 30 min.

#### Environmental control effects

Based on the daily variation data for temperature, humidity, and CO_2_ in the climatic chamber under different treatments, the coefficient of variation of each variable’s set and measured values in the chamber was generally consistent (S1 Table). This means that during the environmentally-controlled and simulated operation of the climatic chamber, all operation values fluctuated around the set value and met the control requirements.

#### Seed and cultivation

The test material was the seed variety that is primarily used in Jingzhou, known as “Liang You 287”. The seeds were soaked in clean water with 3–5 g of seed disinfectant, and then they were placed in an incubator at about 25°C for 24 hours. Turfy soil and black soil (1:3) were placed into each seedling tray. Then, 5–10 pre-soaked seeds were placed in each hole and pressed gently into the soil. Next, the trays were placed into a 28–30°C incubator to accelerate germination for 48 hours. Fresh paddy soil from the study area was used to cultivate the seedlings. This soil was first sieved and then filled into pots 30 cm deep and 20 cm in diameter. Each filled pot weighed about 20 kg. Each treatment required 15 pots, so with nine treatments, a total of 135 pots were used. After the rice seedlings had three complete leaves and one interior leaf in the 25°C incubator, the seedlings were transplanted into the pots with three holes in the soil of each pot and 2–3 seedlings planted in each hole. Pots were then transferred into the climatic chamber and testing was started. Plants were observed until the end of the growing stage.

Fertilization and watering were conducted as follows. Before transplanting, 2 g of urea and 0.5 g of monopotassium phosphate were applied as base fertilizers in the soil of each pot. At 7 days after transplanting, 0.5 g of urea was applied to each pot. During the panicle differentiation stage, 0.6 g of urea was applied to each pot. Plants were given adequate water during the seedling stage. During the maturation stage, soil moisture was alternated between wet and dry. During the other growth stages, a 5 cm deep shallow layer of water was maintained in the pots. Pots were randomly moved in the chamber every 3 days to reduce the influence of location within the chamber on plant growth.

#### Measurement and analysis

The present study involved measurement of three categories of indices: ecological, physiological and rice quality indices. The measurement frequency and methods were as follows:

(1) Ecological indices. These included measures of plant phenology, height, leaf area, and chlorophyll content. Measures of phenology, based on the process of rice growth, involved measuring the number of days plants remained in six stages as follows: 1) seedling, 2) tillering, 3) elongation, 4) heading and blooming, 5) filling, and 6) maturation stages. The measurements of plant height and leaf area were done by randomly selecting three planting holes for each treatment with three seedlings in each hole and consecutively measuring each seedling 3 times every 3 days during each of the above six stages. Chlorophyll content was measured in the second complete leaf (off the leaf vein) from the bottom of the plant in a different direction every 7 days, using a hand-held portable SPAD-502 analyzer (Minolta Camera Co. Ltd., Osaka, Japan).
(2) Physiological indices. These included measures of soluble sugar (SS), soluble protein (SP), malondialdehyde (MDA), proline (Pro) and superoxide dismutase (SOD), and were measured by referring to the measurement methods in -Li [12]. Physiological indices were measured in the seedling, heading, and filling stages. For each treatment, 0.5 g of rice leaf was used as follows: thiobarbituric acid, toluene, coomassie brilliant blue, and ahthrone colorimetric methods were used to measure MDA, Pro, SP, and SS, respectively, and the specific SOD measurement method was referred to in the literature [10]; all measurements were repeated three times, and an average value was calculated and used.
(3) Rice quality. This included measurements of the head rice rate, chalky rice rate, amylose content, and protein content. After the rice matured, 1000 grains were tested for each treatment. These samples were sent to the Rice Quality Testing Center of the Chinese Academy of Agricultural Sciences to measure the relevant indices.

#### Statistical analysis methods

Excel and SPSS were employed to prepare the figures and process the data, with the Duncan range method for testing significance (*p* < 0.05 was considered significant).

## Results and discussion

### Effects of increased atmospheric CO_2_ levels and high temperature on rice phenology

Phenology can reflect the ability of a plant to adapt to environmental change. The results of the present study showed that the number of days in the entire growth period for rice was shortened as CO_2_ and temperature simultaneously increased. The total number of days the entire growth period under the M and H treatments decreased by 12.0% and 22.1%, respectively, when compared with that of CK treatment (Table 1). When comparing all growth stages, the tillering and filling stages were particularly sensitive to the simultaneous increase of CO_2_ and temperature. In the tillering stage, the number of days under the M and H treatments decreased by 18.8% and 31.3%, respectively, when compared with that of the CK treatment. In the filling stage, the number of days under the M and H treatments decreased by 26.2% and 31.0%, respectively, when compared with under the CK treatment.

**Table 1:** Response of rice phenology in different growing periods under different treatments of simultaneously increased CO_2_ and temperature.

Treatment	Seedling stage (d)	Tillering stage (d)	Elongation stage (d)	Heading stage (d)	Filling stage (d)	Maturation stage (d)	Whole growth stage (d)
CK	20.7	16.0	26.3	12.0	14.0	30.0	119.0
M	18.7	13.0	23.0	11.0	10.3	28.7	104.7
H	17.3	11.0	20.3	8.7	9.7	25.7	92.7
Variability between M and CK (%)	(-)9.7	(-)18.8	(-)12.7	(-)8.3	(-)26.2	(-)4.4	(-)12.0
Variability between H and CK (%)	(-)16.1	(-)31.3	(-)22.8	(-)27.8	(-)31.0	(-)14.4	(-)22.1

Note: The number of days in this table represents the days of different growth stages after rice transplanting; minus represents shortening; CK stands for the control treatment, 400 μmol·mol^−1^ +0°C; M stands for moderate treatment, 550 μmol·mol^−1^ +2°C; H stands for intensive treatment, 650 μmol·mol^−1^ +4°C.

### The response of ecological indices to increased atmospheric CO_2_ levels and high temperature

#### Leaf area

**Fig 1:**
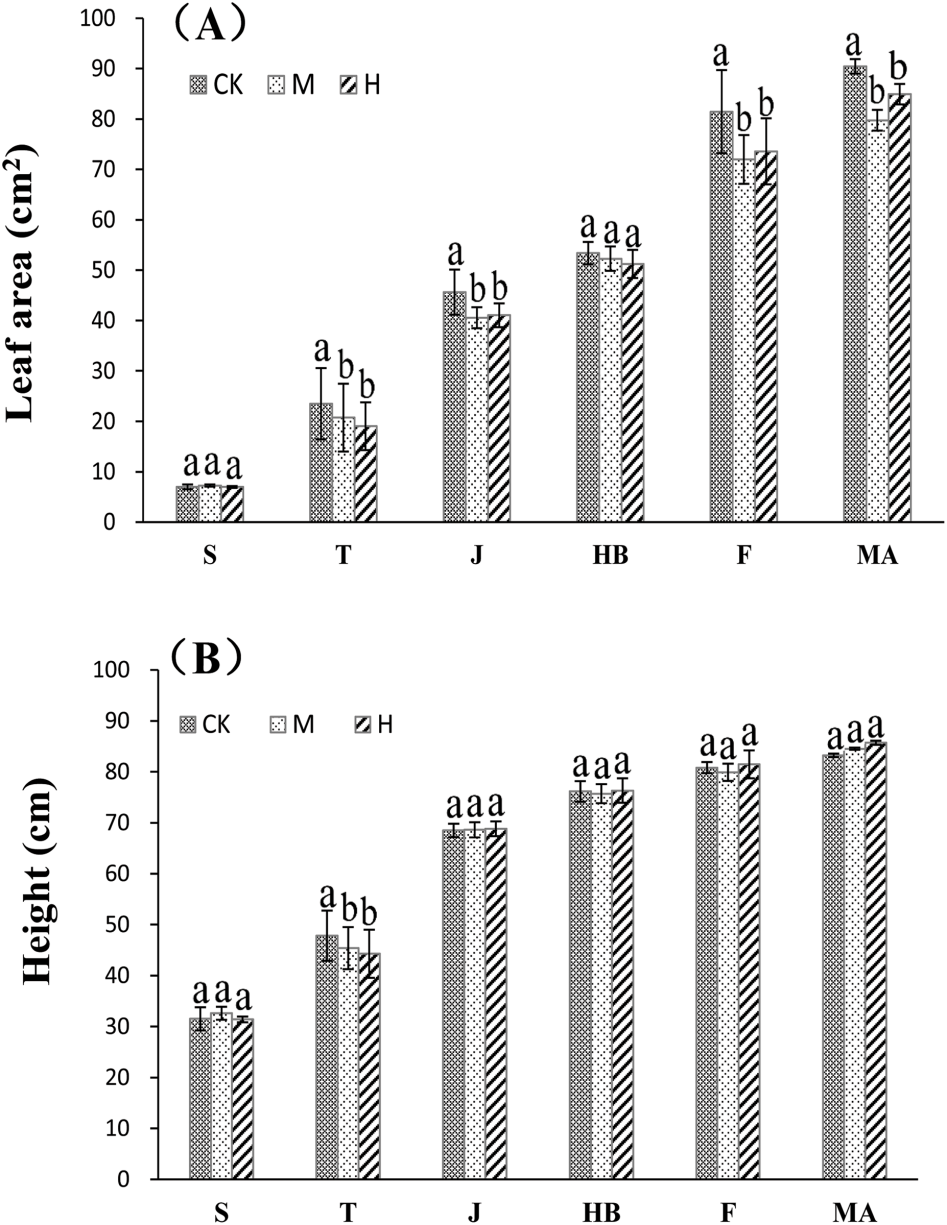
Effects of increased atmospheric CO_2_ levels and high temperature on leaf area (A) and plant height (B). Note: S, T, J, HB, F and MA stand for the seeding, tillering, jointing, heading and blooming, as well as the filling and maturation stages, respectively; a, b and c indicate significant differences at the 0.05 level in the same growth stage.

The variation in rice leaf area is closely related to dry matter accumulation process and further influenced rice yield. The results of the present study showed that as the rice growing stage was extended, leaf area gradually expanded as CO_**2**_ and temperature simultaneously increased (Fig 1A). For the variation of leaf area under different treatments in various growth stages, in the seedling as well as the heading and blooming stages, leaf area did not differ obviously under the CK, M, and H treatments. In the tillering stage, leaf area under the M and H treatments decreased by about 19.15% when compared with that of the CK treatment. In the elongation, filling, and maturation stages, leaf area under the M and H treatments significantly decreased about 10.96%, 11.67%, and 10.74% when compared with that under the CK treatment, respectively. This indicated that an increase of CO_**2**_ and temperature slightly inhibited the development of leaf area.

#### Plant height

Plant height was an important feature closely related to rice yield. Plant height increased over time as the rice growing stage extended and as CO_**2**_ and temperature simultaneously increased. However, after the elongation stage, the increase in plant height slowed and peaked during the maturation stage. The results of the present study showed (Fig 1B) that in tillering stage, the difference between both the M and H treatments were obvious with compared with the CK treatment, with an increase of about 19.94% in both those treatments; that is, the difference between the M and H treatments was not obvious. In other growth stages, no obvious difference was observed between the CK, M, and H treatments, which indicated that the tillering stage of the rice used in the study was the stage that was most sensitive to a simultaneous increase of CO_**2**_ and temperature.

### Effects of increased atmospheric CO_2_ levels and high temperature on physiological indices in key growth stages

#### Malondialdehyde (MDA)

**Fig 2:**
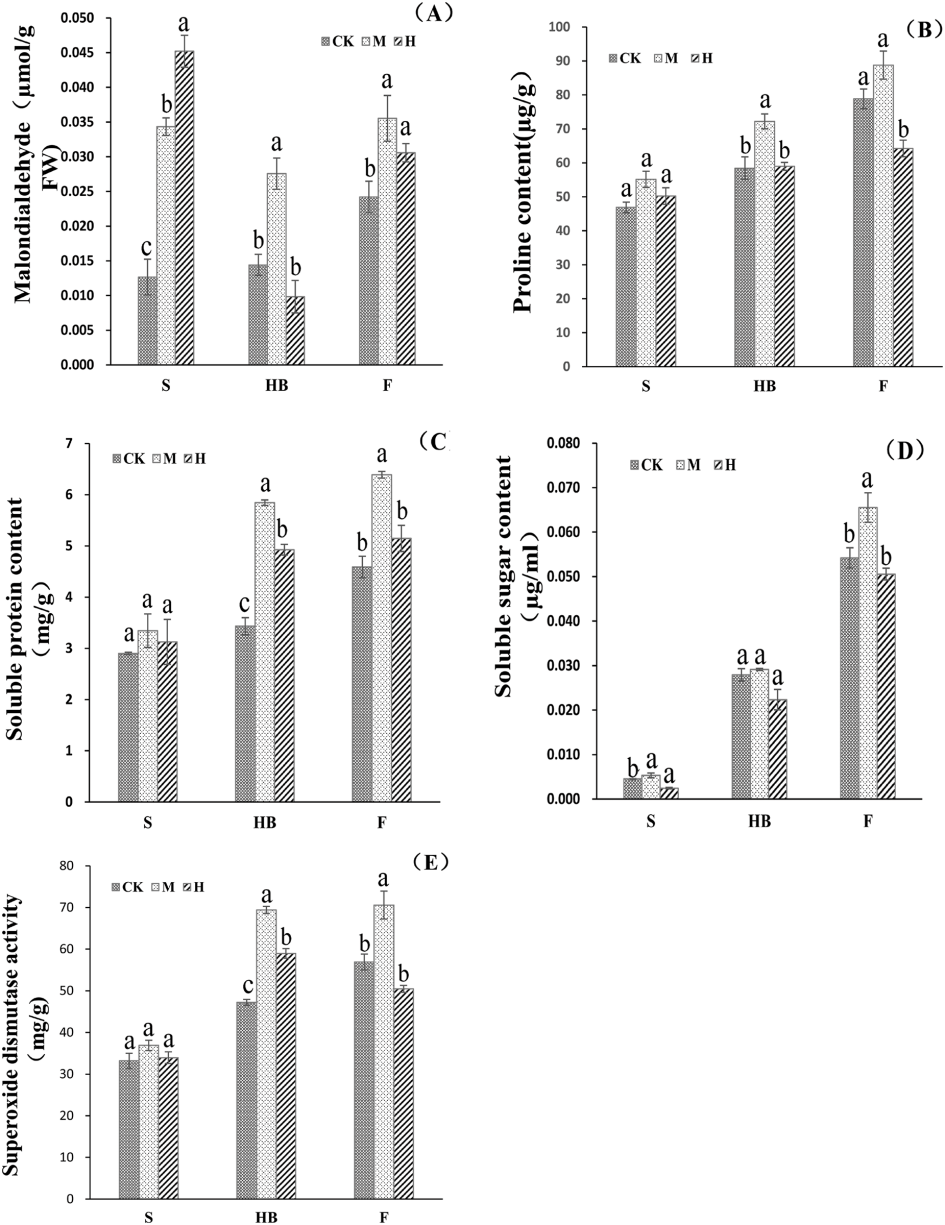
Changes of physiological indices of resistance to stress under the condition of increased atmospheric CO_2_ levels and high temperature in key growth stages. Note: (a)–(e) represents MDA, Pro, SP, SS and SOD, respectively; S, HB, and F stand for the seeding, heading and blooming, as well as the filling stage, respectively. Note: The number of days represents the days of different growth stages after rice transplanting; - symbol represents shortening; CK stands for control treatment, 400 μmol·mol^−1^ +0°C; M stands for moderate treatment, 550 μmol·mol^−1^ +2°C; H stands for intensive treatment, 650 μmol·mol^−1^ +4°C. Note: a, b and c indicate significant differences at the 0.05 level in the same growth stage.

MDA is the final product of membrane lipid peroxidation within rice plants. MDA content reflects the degree of cell membrane lipid peroxidation. As the level of cell membrane lipid peroxidation increases, the structural integrity of the cell membrane tends to degrade. The results of the present study showed (Fig 2A) that for different treatments in the same stage, as the levels of CO_**2**_ and temperature increased simultaneously, the MDA content in seedling stage increased significantly. In addition, the MDA content in heading and filling stages initially increased and subsequently decreased. For the same treatment in different stages, as the length of each growth stage extended, MDA under the CK treatment increased, while MDA under the M and H treatments initially decreased and subsequently increased. However, the difference in MDA levels between the CK and M treatments was not significant. This finding indicated that an increase of CO_**2**_ and temperature tended to impair the structure of the leaf membrane in rice. Meanwhile, as the growing stage extended, this type of leaf impairment decreased. One can conclude that the plant’s physiological properties may allow the plant to develop and adapt to this type of change.

#### Proline content (Pro)

Proline content would increase in the state of plant cell exsiccosis. The present study showed (Fig 2B) that for the same treatment in different stages of rice growth, Pro content in rice leaves under the CK, M, and H treatments increased as the growing stages extended and Pro content under the M treatment peaked in various growth stages. No significant difference in Pro content was observed for each respective stage under the CK and H treatments. Overall, Pro content under CK, M, and H treatments peaked in the filling stage, which indicated that the rice leaves in filling stage were prone to have a relatively insufficient supply of water.

#### Soluble protein (SP)

SP serves as the main form of plant nitrogen. An increase in nitrogen will reflect a decrease of plant synthetic and metabolic ability. The results of the present study showed (Fig 2C) that for the same stage under different treatments, SP content in seedling, heading, and filling stages simultaneously increased along with CO_**2**_ and temperature. Specifically, the SP content tended to initially increase and then increase, and peaked under the M treatment; this indicated that a moderate simultaneous increase of CO_**2**_ and temperature may facilitate the decomposition of SP and increase the SP content in the leaves. Meanwhile, an intensive simultaneous increase of CO_**2**_ and temperature may decrease the leaves’ metabolic ability with SP decreasing accordingly. For the same treatment at different stages, the SP content under the CK, M, and H treatments gradually increased as the growing stage extended; however, the increase under the M and H treatments in heading and filling stages was less than that under the CK treatment. This finding indicated that under intensive treatment in the heading stage, the decomposition of SP was accelerated, which was sustained until the metabolic and decomposing ability weakened after filling stage and the decomposition rate of nutrient substances slowed.

#### Soluble sugar (SS)

Soluble sugar serves as the main form of plant carbon nutrition, and provides energy-containing substances for the plant; therefore, SS plays an important role in sustaining the stability of plant proteins. Soluble sugar that accumulates in the plant cell participates in osmotic protection, osmotic adjustment, carbon storage and the removal of reactive oxygen. The results of the present study showed (Fig 2D) that for the same stage under different treatments, the SS content under the CK, M, and H treatment in the seedling stage did not vary significantly. The SS levels under the CK and M treatments in the heading stage did not vary significantly, while SS under the H treatment significantly decreased. The level of SS under the M treatment in the filling stage was significantly higher than that under the CK and H treatments. This finding indicated that in seedling and heading stages, a simultaneous increase of CO_**2**_ and temperature did not influence the ability of a rice leaf cell to protect itself. However, during the filling stage, a moderate simultaneous increase of CO_**2**_ and temperature would facilitate the decomposition of SS, enhance leaf cell membrane infiltration capacity, and increase the accumulation of carbon nutrition.

#### Superoxide dismutase (SOD)

SOD is one of the most effective antioxidases, and it could remove potentially dangerous superoxide anions and hydrogen peroxide allowing it to help relieve the impairment of plant cells. The results of the present study showed (Fig 2E) that for the same stage under different treatments, SOD vitality did not differ significantly during the seedling stage. During the heading and filling stages, SOD vitality significantly increased and subsequently decreased as CO_**2**_ and temperature simultaneously increased. For the same treatment at different stages, as the growth stage extended, SOD vitality under the CK, M, and H treatments gradually increased. That is, SOC vitality increased until the end of the heading stage and then remained constant, initially increasing and subsequently decreasing. This finding indicated that in the seedling stage, a simultaneous increase of CO_**2**_ and temperature did not impair the rice leaves. However, as the growth stage and handling time extended, the impairment to leaves was gradually worsened in the heading stage and stopped increasing until the end of the filling stage.

### Effects of increased atmospheric CO_2_ levels and high temperature on rice quality

**Fig 3:**
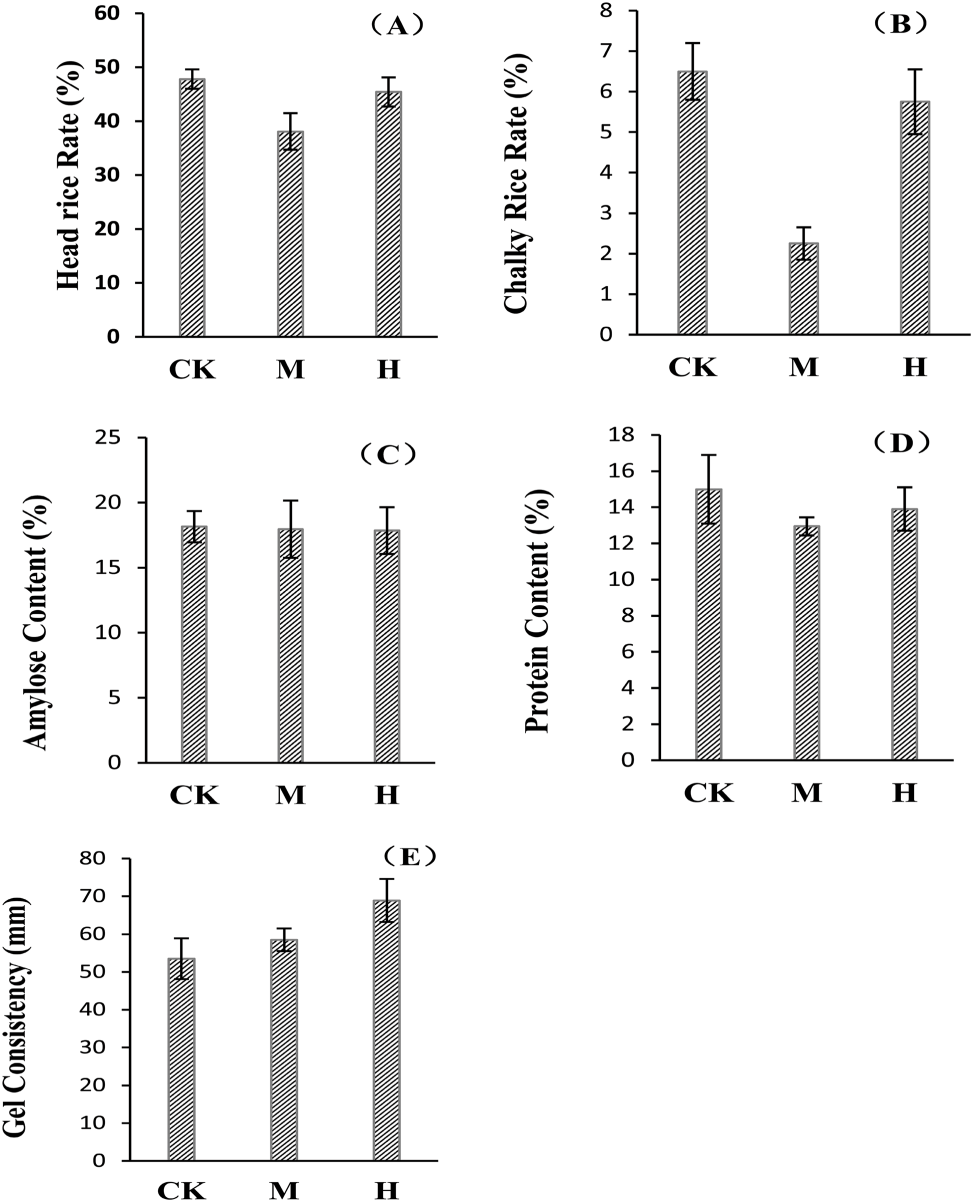
Changes of rice quality indices under the condition of increased atmospheric CO_2_ levels and high temperature. (A)–(E) represent the Head Rice Rate, Chalky Rice Rate, Amylose Content, Protein Content and Gel Consistency, respectively.

The head and chalky rice rates are important quality features of commercially processed rice. The results of the present study showed (Fig 3A) that head rice rate initially decreased slightly and subsequently increased as CO_2_ and temperature increased. The head rice rate under the CK treatment was 25.46% and 5.29% higher than that under the M and H treatments, respectively. The chalky rice rate under the CK treatment was higher than that under the M and H treatments. The chalky rice rate was lowest under the M treatment, only 2.3%, which was 64.62% lower than that under the CK treatment (Fig 3B).

Amylose and protein content of rice serve as important indices of rice nutritional quality. Amylose content did not vary significantly under the CK, M, and H treatments (Fig 3C); as CO_2_ and temperature increased, protein content decreased. Protein content under the CK treatment was the highest, 15%. Protein content under the M treatment was the lowest, 14% lower than that under the CK treatment. Protein content under the H treatment decreased to 7.33% of that under the CK treatment (Fig 3D).

Gel consistency serves as an important index of the edibility and quality of rice. Gel consistency is the length of gel after gelatinization and cooling of rice starch and is used to indicate the recovery of starch after gelatinization and cooling. A higher value meant the rice would be softer after being steamed or boiled, with better quality. In the present study, the rice gel consistency gradually increased as CO_2_ and temperature simultaneously increased (Fig 3E), which improved the edibility and quality of rice.

## Conclusions

### Responses of ecological indices to increased CO_2_ and temperature

Phenology, an important ecological feature of rice, indicates whether the plant developed a growth and development pattern that allowed it to adapt to the periodic variation of temperature conditions over a long time [11]. Previous studies showed that an increase in atmospheric CO_2_ and temperature would accelerate the growth process of plants [13–14], and high levels of CO_2_ would cause the rice to enter the blooming stage earlier, while high temperature would cause rice to have an earlier heading stage [15]. Based on the test results of a simultaneous increase in CO_2_ and temperature in the present study (Table 1), these increases shorten the entire growth stage by 26 days, with higher proportions occurring in the elongation and filling stages. This finding indicates that a simultaneous increase of CO_2_ and temperature would make rice plants have an earlier leafing and blooming stage while the entire growth stage would be shortened. This phenomena may be caused by the acceleration of material migration in these two rice growth stages under the high level of CO_2_ enrichment and temperature increasing [16, 17], which is similar to findings of previous studies.

Leaf area is a basic index used to measure the transformation of light energy into chemical energy within plant leaves [17]. Previous studies have shown that leaf area enlarged as the availability of atmospheric CO_2_ increased, and an increase of CO_2_ had a certain manurial effect on the growth of rice leaf area [18]. However, leaf area declined as temperature rose, and increased temperature had a certain inhibiting effect on the growth of rice leaf area [15]. The results of the present study showed that with an exception of the seedling stage, the leaf area decreased to a different extent as CO_2_ and the temperature increased in other growth stages under different treatments (Fig 1A). The reason may be that under the M and H treatments, the temperature rose to 30–40°C, which exceeded the optimum temperature range for rice growth (usually 35°C), causing high temperature stress to inhibit the generation of photosynthates [17], and reduced dry matter accumulation, which offset the manurial effect of increased CO_2_ on rice leaf area growth.

The plant height was proportional to yield and quality within a certain range. For high-yielding rice varieties, the internode length under the ear of rice plus the length of the ear equaled to 45–50% of the plant height [14, 18], with large-size grain, which influenced the appearance and quality of rice. According to the results of previous studies, rice plant height significantly increased after an increase of CO_2_ and temperature [19]. The present study found similar results with a simultaneous increase of CO_2_ and temperature (Fig 1B). Probably because the effects of increased CO_2_ on plant height formative factors were an independent genetic expression, the inner relationship was not prone to be influenced by high temperature.

### Response of rice physiological indices to the increase of CO_2_ and temperature

Plants require malondialdehyde (MDA) for plant survival. MDA is an oxygen-containing substance with strong oxidizing capacity that is generated when oxidoreduction was incomplete in aerobic metabolism; a high concentration of oxygen-containing substances may cause strong harm to plant cells [20]. When environmental stress acted on the plant for a long time, a plant would generate membrane lipid peroxidation and accumulate MDA, the product of membrane lipid peroxidation; therefore, more MDA meant the plant was obviously experiencing stress [21]. The results of the present study showed (Fig 2A) that in the seedling, heading and filling stages, levels of MDA were not significantly different in rice leaves, and the MDA level gradually decreased as CO_2_ and temperature simultaneously rose. This finding indicated that the treatments in the present study did not cause stress to plant leaves. This was mainly because in the present study, after the temperature rose, the range of variation in the maximum temperature in the rice growing stage was 28–34°C (S1 Table), which didn’t cause obvious high temperature stress. In addition, the manurial effect of CO_2_ may offset some impairment to membrane lipids.

Proline (Pro) is an important organic osmotic substance in the plant. Almost all adverse situations, such as drought, high temperature, low temperature, freezing, malnutrition, disease, air pollution and so on, would cause proline accumulate in the plant [22]. Pro could maintain the protoplasm and environmental osmotic balance, prevent water loss and also serves as a nitrogen reserve substance providing the energy needed to recover growth [23]. To a certain extent, Pro content in the crop reflected the water content in the crop and Pro content increased in case of water shortage [22]. The results of the present study showed (Fig 2B) that proline content significantly increased during the heading stage but under 650 μmol·mol^−1^ +4°C treatment, the proline content change was mostly lower than that under the CK treatment. This finding indicated that the interaction effect of high CO_2_ and temperature rise didn’t cause serious stress to plant growth, mainly because high CO_2_ increased the water use efficiency of the plant, thereby decreasing the Pro content in the rice leaves [24].

Soluble protein (SP) was the main form of plant nitrogen, as well as the main material used to regulate and control plant metabolism. The change in SP reflected the plant synthetic and metabolic ability, so SP has usually been used as an important index to measure the degree of leaf aging [25]. The results of the present study showed (Fig 2C) that SP content initially decreased and then increased in both the seedling and filling stages, and gradually decreased in heading stage. SP in these three stages did not vary widely, perhaps because the daily average temperature increased faster in the heading stage, caused premature leaf aging, and accelerated the function of leaves synthesizing protein and sending it to the grain, thereby making the SP content in the heading stage decrease rapidly [26].

Soluble sugar serves as the main form of plant carbon nutrition, which provided energy substances for the plant and played an important role in sustaining the stability of plant protein [27]. Soluble sugar that accumulated in the plant cell participated in osmotic protection, osmotic adjustment, carbon storage, and the removal of reactive oxygen; the variation in SS content is closely related to the photosynthetic ability of rice leaves [17, 28]. Previous studies have shown that a high CO_2_ concentration would increase the SS content in plant leaves, but there was no agreement on the effect of high temperature on SS. Some studies have been thought to show that high temperature stress would also increase the SS in the plant; SS mainly maintained protein hydrature, reduced the loss of protoplasm water, and was helpful for balancing the osmotic potential between vacuole and cell, thereby decreasing the impairment of lipid membrane and enhancing the plant’s ability to tolerate heat [27]. In the present study, compared to moderate treatment, under 650 μmol·mol^−1^+4°C treatment, simultaneous increase of CO_2_ and temperature increased the SS in the rice leaves in heading and filling stages, while in the seedling stage, the SS content decreased as the treatment intensified (Fig 2D). This occurred because a simultaneous increase of CO_2_ and temperature facilitated photosynthesis in rice leaves, increased the rate of carbohydrate synthesis and conversion, and thereby enhanced the energy source for SS content in the leaves [26, 29].

Superoxide dismutase (SOD) serves as a protective enzyme system formed in the cell to defend the impairment of reactive oxygen in the biological evolution process; it could remove potentially dangerous superoxide anions and hydrogen peroxide, relieve the impairment to plant cells, and control lipid oxidation. A higher SOD value meant the plant experienced higher stress levels [30]. The results of the present study showed (Fig 2E) that the overall SOD activity significantly increased in the heading stage and was higher than that under CK treatment, while overall SOD activity in both the seedling and filling stages was lower than that under the control treatment. This occurred mostly because the daily average temperature increase in the heading stage was more than that of seedling and filling stages and the high temperature stress reduced the level of SOD activity [24].

### Response of rice quality to increases in CO_2_ and temperature

Rice quality was mainly reflected by appearance, nutritional and edible qualities of rice; while appearance included the head rice rate and chalky rice rate, nutritional quality included amylose and protein content, and edible quality included gel consistency. These characters directly influenced the overall rice quality and rice sales [31]. The present study showed that an increase of CO_2_ and temperature caused head rice rate and chalky rice rate initially to decrease and then increase (Fig 3A and 3B), but these were generally lower than that under control treatment (Fig 3C). This finding indicated that high temperature and CO_2_ impaired the appearance quality of rice and was similar with the results of Xie et al. (2009) [13]. For the nutritional quality, the amylose content had no significant difference under different treatments in the present study; however, protein content under high temperature and CO_2_ treatment was lower than that under the control treatment (Fig 3D). Gel consistency under high temperature and CO_2_ treatment gradually increased, which meant the softness of edible quality improved.

In conclusion, under increased atmospheric CO_2_ levels and high temperature, the rice’s phenology shortened, the leaves received certain stress, and the appearance and processing quality slightly worsened.

## Supporting information

S1 Table Diurnal variation of temperature (T), relative humidity (RH), and CO2 concentration in climatic chambers. Note: (1) SV is set value, MV is monitoring value; (2) The set values of temperature and moisture under CK (400 μmol·mol^−1^) treatment are based on the real observation data at Jing Zhou experiment site in 2013; M stands for moderate treatment, 550 μmol·mol^−1^ +2°C; H stands for intensive treatment, 650 μmol·mol^−1^ +4°C. (3) SD is standard deviation, MN is mean value, and CV is coefficient of variation, CV = SD/MN×100.
(DOCX)
