# Supplementary material for: Effects of increased levels of atmospheric CO2 and high temperatures on rice growth and quality
Source: PLoS One. 2017 Nov 16;12(11):e0187724. doi: 10.1371/journal.pone.0187724 (PMC5690611; doi:10.1371/journal.pone.0187724)
Supplement: S1 Table — Note: (1) SV is set value, MV is monitoring value; (2) The set values of temperature and moisture under CK (400 μmol·mol−1) treatment are based on the real observation data at Jing Zhou experiment site in 2013; M stands for moderate treatment, 550 μmol·mol−1 +2°C; H stands for intensive treatment, 650 μmol·mol−1 +4°C. (3) SD is standard deviation, MN is mean value, and CV is coefficient of variation, CV = SD/MN×100. (DOCX) [file pone.0187724.s001.docx]

**Supporting information**

S1 Table. Diurnal variation of temperature (T), relative humidity (RH), and CO_2_ concentration in climatic chambers

| **Treatment** | **Indices** | | **Time** | | | | | | | | | | | | **SD** | **MN** | **CV (%)** |
| --- | --- | --- | --- | --- | --- | --- | --- | --- | --- | --- | --- | --- | --- | --- | --- | --- | --- |
|  |  |  | **0:00** | **2:00** | **4:00** | **6:00** | **8:00** | **10:00** | **12:00** | **14:00** | **16:00** | **18:00** | **20:00** | **22:00** |  |  |  |
| CK | Temperature (°C) | SV | 24.8 | 24.5 | 24.6 | 24.7 | 25.5 | 27.0 | 28.5 | 28.8 | 27.9 | 26.9 | 26.1 | 25.4 | 1.6 | 26.2 | 6.0 |
|  |  | MV | 25.0 | 24.7 | 24.8 | 25.0 | 25.8 | 27.5 | 29.0 | 29.3 | 28.1 | 27.1 | 26.2 | 25.6 | 1.7 | 26.5 | 6.3 |
|  | Relative Humidity (%) | SV | 87.6 | 88.6 | 87.8 | 87.0 | 83.7 | 77.7 | 71.7 | 70.5 | 74.3 | 78.1 | 81.4 | 84.3 | 6.5 | 81.1 | 8.0 |
|  |  | MV | 85.3 | 87.0 | 86.5 | 85.2 | 82.6 | 76.3 | 72.5 | 71.5 | 75.7 | 79.5 | 82.3 | 84.3 | 5.5 | 80.7 | 6.8 |
|  | CO_2_ (molmol^-1^) | SV | 400 | 400 | 400 | 400 | 400 | 400 | 400 | 400 | 400 | 400 | 400 | 400 | 0 | 400 | 0 |
|  |  | MV | 440 | 447 | 457 | 458 | 438 | 395 | 341 | 349 | 395 | 453 | 459 | 456 | 43 | 424 | 10.19 |
| M | Temperature (°C) | SV | 26.8 | 26.5 | 26.6 | 26.7 | 27.5 | 29.0 | 30.5 | 30.8 | 29.9 | 28.9 | 28.1 | 27.4 | 1.6 | 28.2 | 5.5 |
|  |  | MV | 26.7 | 26.3 | 26.4 | 26.6 | 27.5 | 29.0 | 30.5 | 30.9 | 29.7 | 28.7 | 27.9 | 27.2 | 1.6 | 28.1 | 5.8 |
|  | Relative Humidity (%) | SV | 87.6 | 88.6 | 87.8 | 87.0 | 83.7 | 77.7 | 71.7 | 70.3 | 74.6 | 78.1 | 81.4 | 84.3 | 6.5 | 81.1 | 8.0 |
|  |  | MV | 73.3 | 72.6 | 72.4 | 73.0 | 70.9 | 69.5 | 68.8 | 68.0 | 71.2 | 72.0 | 72.1 | 72.0 | 1.7 | 71.3 | 2.4 |
|  | CO_2_ (molmol^-1^) | SV | 550 | 550 | 550 | 550 | 550 | 550 | 550 | 550 | 550 | 550 | 550 | 550 | 0 | 550 | 0 |
|  |  | MV | 549 | 550 | 554 | 559 | 553 | 546 | 532 | 533 | 560 | 555 | 549 | 547 | 9 | 549 | 1.59 |
| H | Temperature (°C) | SV | 28.8 | 28.5 | 28.6 | 28.7 | 29.5 | 31.0 | 32.5 | 32.8 | 31.9 | 30.9 | 30.1 | 29.4 | 1.6 | 30.2 | 5.2 |
|  |  | MV | 28.8 | 28.5 | 28.6 | 28.8 | 29.6 | 31.1 | 32.6 | 33.0 | 31.9 | 30.9 | 30.1 | 29.5 | 1.6 | 30.3 | 5.3 |
|  | Relative Humidity (%) | SV | 87.6 | 88.6 | 87.8 | 87.0 | 83.7 | 77.7 | 71.7 | 70.5 | 74.3 | 78.1 | 81.4 | 84.3 | 6.5 | 81.1 | 8.0 |
|  |  | MV | 69.1 | 68.8 | 68.8 | 67.6 | 66.4 | 62.7 | 61.0 | 60.1 | 60.7 | 63.6 | 65.5 | 65.7 | 3.3 | 65.0 | 5.1 |
|  | CO_2_ (molmol^-1^) | SV | 650 | 650 | 650 | 650 | 650 | 650 | 650 | 650 | 650 | 650 | 650 | 650 | 0 | 650 | 0 |
|  |  | MV | 700 | 700 | 700 | 699 | 694 | 690 | 699 | 688 | 701 | 696 | 700 | 700 | 4 | 697 | 0.6 |

1. Note: (1) SV is set value, MV is monitoring value; (2) The set values of temperature and moisture under CK (400 μmol⋅mol^−1^) treatment are based on the real observation data at Jing Zhou experiment site in 2013; M stands for moderate treatment, 550 μmol⋅mol^−1^ +2°C; H stands for intensive treatment, 650 μmol⋅mol^−1^ +4°C. (3) SD is standard deviation, MN is mean value, and CV is coefficient of variation, CV=SD/MN×100.
